# Preparation and Properties of New Co-Crystals of Ibandronate with Gluco- or Galactopyranoside Derivatives †

**DOI:** 10.3390/molecules15128973

**Published:** 2010-12-08

**Authors:** Zbynek Oktabec, Jiri Kos, Zuzana Mandelova, Lenka Havelkova, Tomas Pekarek, Anna Rezacova, Lukas Placek, Marcela Tkadlecova, Jaroslav Havlicek, Jiri Dohnal, Josef Jampílek

**Affiliations:** 1 Zentiva k.s., U Kabelovny 130, 102 37 Prague 10, Czech Republic; 2 Department of Inorganic and Organic Chemistry, Faculty of Pharmacy in Hradec Kralove, Charles University in Prague, Heyrovskeho 1203, 500 05 Hradec Kralove, Czech Republic; 3 Department of Chemical Drugs, Faculty of Pharmacy, University of Veterinary and Pharmaceutical Sciences, Palackeho 1/3, 612 42 Brno, Czech Republic

**Keywords:** ibandronate, d-glucopyranosides, d-galactopyranosides, co-crystals, FT-NIR, FT-Raman, CP/MAS NMR, PAMPA

## Abstract

Mixtures of ibandronate monosodium salt with eleven gluco- and/or galacto-pyranoside derivatives as counterions were designed to prepare co-crystals with improved intestinal absorption. In general, gastrointestinal absorption of bisphosphonates after oral administration is approximately 1%. Co-crystals were generated by means of thermodynamically and/or kinetically controlled crystallization processes. Seventy-seven prepared samples were analyzed by means of FT-NIR, FT-Raman spectrometry and solid state NMR spectroscopy. New entities of ibandronate monosodium salt with phenyl-β-d-galactopyranoside were found and characterized. The absorption of these potential new co-crystals was investigated by means of PAMPA experiments. In the present study the relationships between the chemical structures of the studied compounds required for co-crystal generation are discussed.

## 1. Introduction

Bisphosphonates (BPs) are the most widely used and the most effective bone resorption inhibitors currently available for treatment of Paget’s disease, tumor-associated bone disease and osteoporosis. All BPs have high affinity for bone mineral as a consequence of their P-C-P backbone structure, which allows chelation of calcium ions [1]. Following release from bone mineral during acidification by osteoclasts, BPs appear to be internalized specifically by osteoclasts, but not other bone cells [2]. The intracellular accumulation of BP leads to inhibition of osteoclast function due to changes in the cytoskeleton, loss of the ruffled border [2,3] and apoptosis [4,5,6,7]. The ability of BPs to inhibit bone resorption depends on the presence of two phosphonate groups in the P-C-P structure, which appear to be required for interaction with a molecular target in the osteoclast as well as for binding bone mineral [8,9,10].

**Figure 1:**
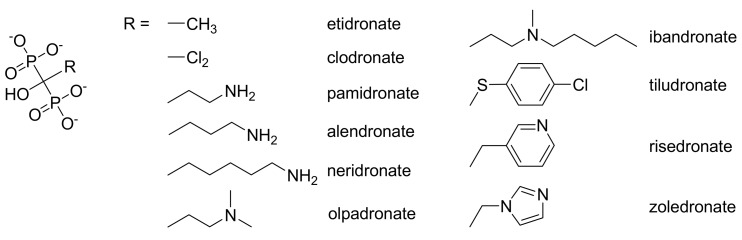
Structures of bisphosphonates.

Bisphosphonates are a group of drugs that are widely used in practice as pyrophosphate analogues (see the general structure in Figure 1). There are several injectable bisphosphonates: etidronate (Didronel^®^), pamidronate (Aredia^®^) and zoledronate (Zometa^®^), which may be administered every three months or yearly. Ibandronate in contrast with alendronate (Fosamax^®^) and risedronate (Actonel^®^), which can be taken daily or weekly, is the only oral revolution bisphosphonate that is approved to be taken monthly [11]. Oral bioavailability of these bisphosphonates is very low (their gastrointestinal absorption is about 1%) due to their high hydrophilicity [12].

In general, structural modifications are the best way to improve permeability: *i*) replacement of ionisable groups by non-ionizable groups; *ii*) increase of lipophilicity; *iii*) isosteric replacement of polar groups; *iv*) esterification of carboxylic acid; *v*) reduction of hydrogen bonding and polarity; *vi*) reduction of size; *vii*) addition of a nonpolar side chain; *viii*) preparation of prodrugs. Generally these strategies are based on a few fundamental concepts: reduction of ionizability, increase of lipophilicity, reduction of polarity or reduction of hydrogen bond donors or acceptors. Thus, it is important to assess permeability early and to build permeability improvement into the synthetic plan from the beginning. This could rescue a chemical series that has great potential and improve drug exposure in animal pharmacology and pharmacokinetic studies [13].

Formulation is other strategy for improving permeability and bioavailability. For example, permeability enhancers, surfactants or pharmaceutical complexing agents can be used in the oral dosage form [13].

The application of co-crystal technologies has only recently been recognised as a way to enhance solubility, stability and the intellectual property (IP) position with respect to the development of active pharmaceutical ingredients (APIs). Unlike salt formation, co-crystallisation does not rely on ionisation of the API and the counterion to make a solid. Instead, both components utilise prominent intermolecular interactions, such as hydrogen bonding, to combine and yield a uniform crystalline material. Combining an API with a pharmaceutically acceptable agent in this guest/host manner has become an increasingly attractive route for developing pharmaceutical products. For example, co-crystallisation offers an alternative when salt screening is either unsuccessful or impossible (due to lack of ionisation sites) to improve the physical properties of a drug. Furthermore, exploring the co-crystallisation potential around an API increases the intellectual property protection over a particular drug product, thus reducing the risk of costly litigation and market erosion. A recent development in the field has not only shown co-crystallisation as an alternative to salt studies, but has also shown its combination with salts to yield co-crystals of salts [14]. Co-crystals of API with common pharmaceutical excipients become very important [15,16].

Due to the above mentioned facts, some experiments were designed to prepare co-crystals of ibandronate as a basis for possible super generics. In the present study various mixtures of ibandronate and excipients (more hydrophobic adducts) in different ratios and under various conditions were prepared. All the prepared mixtures (solid compounds) and/or new entities were characterized by means of the Fourier transform near-infrared (FT-NIR) spectrometry [17]. Potential new co-crystals were also characterized by means of the FT-Raman spectrometry and the solid-state NMR spectroscopy. The confirmed potential co-crystals were investigated for their absorption by means of experiments using the Parallel Artificial Membrane Permeation Assay (PAMPA). 

This is a follow-up paper to our previous works [18,19,20] dealing with preparation and characterization of new potential co-crystals of APIs with excipients.

## 2. Results and Discussion

### 2.1. Chemistry

**Figure 2:**
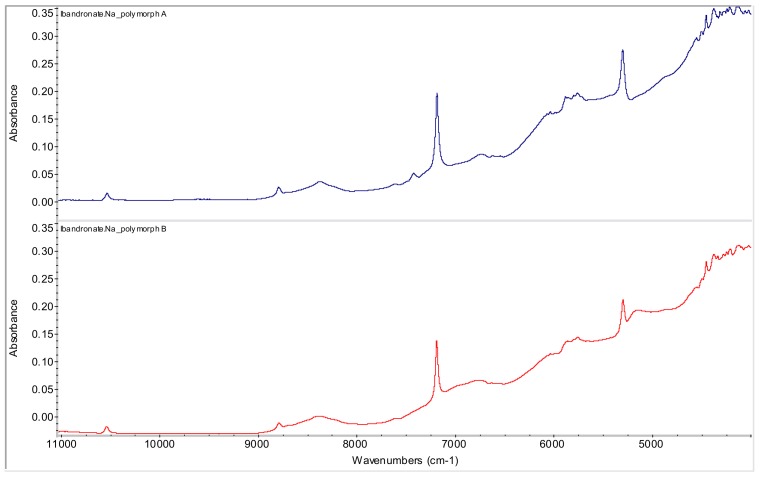
NIR spectra of ibandronate mono-sodium salt polymorph A and starting ibandronate monosodium salt polymorph B.

Ibandronate monosodium salt monohydrate (sodium hydrogen {1-hydroxy-3-[methyl(pentyl)amino]-1-phosphonopropyl}phosphonate, IBN), polymorph B was used as a starting material [21]. It is a white powder, freely soluble in water, practically insoluble in organic solvents. IBN is manufactured by GlaxoSmithKline and Roche Laboratories and is marketed under the trade names Boniva^®^, Bondronat^®^ or Bonviva^®^. Eiermann *et al*. prepared crystalline forms B and A [21,22]. Lifshitz-Liron *et al*. obtained forms C, D, E, F, G, H, J, K, K2, K3, Q, Q1, Q2, Q3, Q4, Q5, Q6, QQ, R, S and T [23]. Muddasani *et al*. prepared polymorphs I and II [24] and Devaraconda *et al*. generated forms III-XXXI [25]. Ibandronate monosodium salt monohydrate polymorph B is the most common in pharmaceutical formulations. NIR spectra of both polymorphs A and B are illustrated in Figure 2.

**Figure 3:**
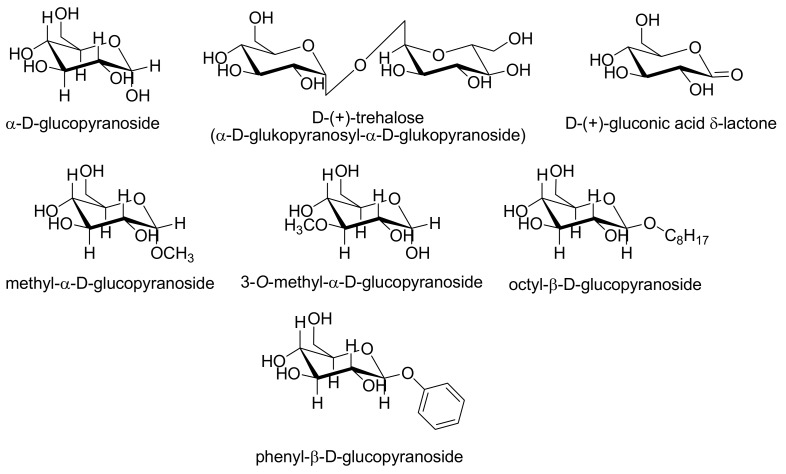
Structure of starting derivatives of glucose used as potential counterions.

**Figure 4:**
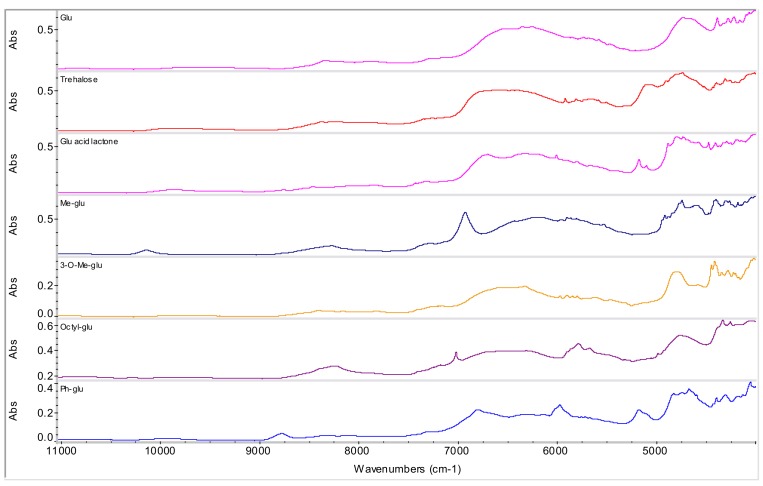
NIR spectra of d-glucopyranose derivatives. (α-d-glucose: Glu, d-(+)-trehalose, d-(+)-gluconic acid δ-lactone: Glu acid lactone, methyl-α-d-glucopyranoside: Me-glu, 3-*O*-methyl-α-d-glucopyranoside: 3-*O*-Me-glu, octyl-β-d-glucopyranoside: Octyl-glu, phenyl-β-d-gluco-pyranoside: Ph-glu).

**Figure 5:**
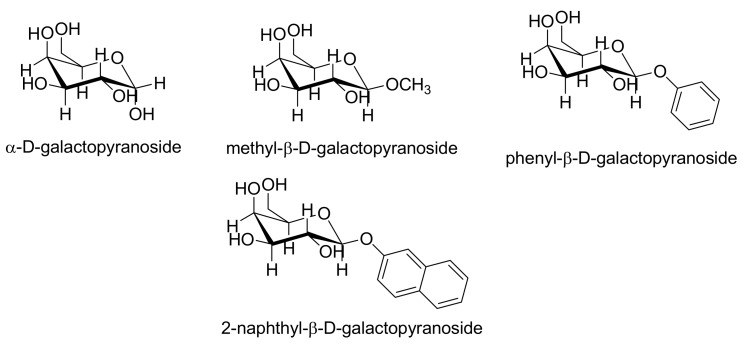
Structure of starting derivatives of galactose used as potential counterions.

**Figure 6:**
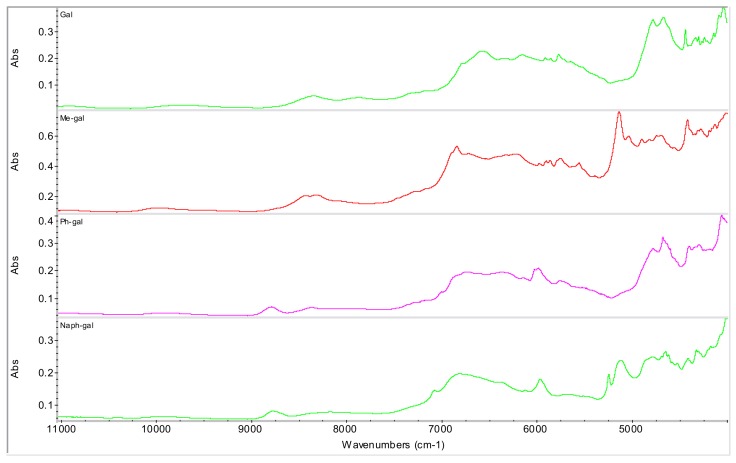
NIR spectra of d-galactopyranose derivatives. (α-d-galactopyranoside: Gal, methyl-β-d-galactopyranoside: Me-gal, phenyl-β-d-galactopyranoside: Ph-gal, 2-naphthyl-β-d-galactopyranoside: Naph-gal).

Various excipients and/or pharmaceutically acceptable agents were evaluated as potential counterions: α-d-glucose, d-(+)-trehalose, d-(+)-gluconic acid δ-lactone, methyl-α-d-glucopyranoside, 3-*O*-methyl-α-d-glucopyranoside, octyl-β-d-glucopyranoside, phenyl-β-d-glucopyranoside (see Figure 3 and Figure 4) and α-d-galactopyranoside, methyl-β-d-galactopyranoside, phenyl-β-d-galacto-pyranoside and 2-naphthyl-β-d-galactopyranoside (see Figure 5 and Figure 6).

The evaluated samples were prepared by means of dissolution of ibandronate mono-sodium salt and gluco- or galactopyranoside derivatives and subsequent reverse obtaining of solid compounds that were characterized using the FT-NIR spectrometry (diffuse reflectance method, DRIFT). The list of generated samples is shown in Table 1.

**Figure 7:**
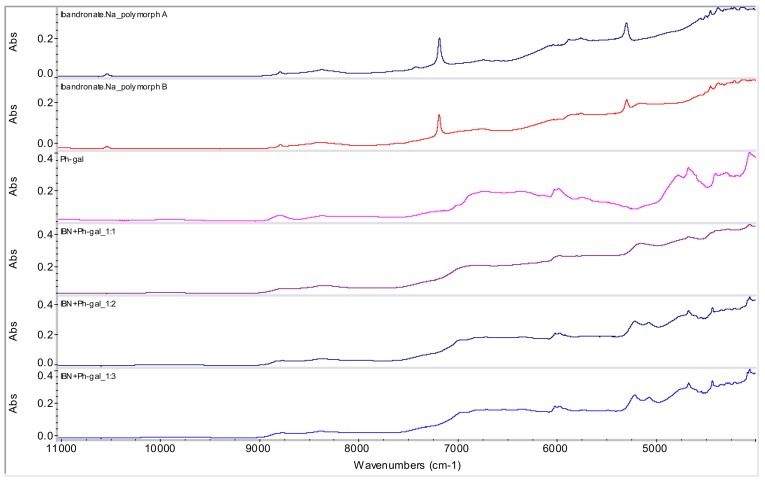
NIR spectra of ibandronate (IBN) monosodium salt forms A and B, phenyl-β-d-galactopyranoside (Ph-gal) and spectra of potential co-crystals of IBN+Ph-gal in ratios 1:1, 1:2 and 1:3 prepared by evaporation at ambient temperature (samples **1**-**3**).

From all tested agents, only phenyl-β-d-galactopyranoside (Ph-gal) yielded interesting products with IBN. Samples of IBN+Ph-gal in ratios 1:1 (**1**), 1:2 (**2**) and 1:3 (**3**) were prepared by mixing and subsequent evaporation at ambient temperature. The spectra are illustrated in Figure 7. In all three samples a change in the spectra can be observed in the 5,300-4,800 cm^-1^ range. The spectra of samples **2** and **3** are very similar, only slightly different from sample **1**, probably due to the lower crystallinity of sample **1**, which causes broader bands in the spectrum. As samples **1**-**3** were prepared in the same way, it can be concluded that increasing Ph-gal concentration influences sample crystallinity.

**Figure 8:**
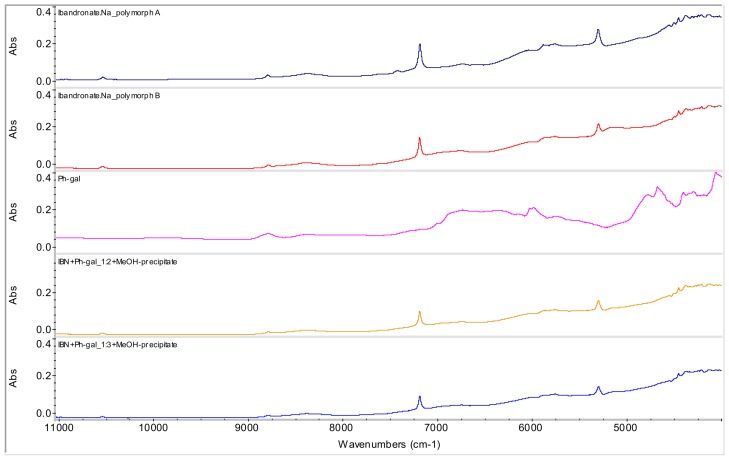
NIR spectra of ibandronate (IBN) monosodium salt forms A and B, phenyl-β-d-galactopyranoside (Ph-gal) and spectra of their mixtures in ratios 1:2 and 1:3 prepared by methanol precipitation (samples **4** and **5**).

Based on the spectra of samples of IBN+Ph-gal in ratios 1:2 (**4**) and 1:3 (**5**) precipitated by MeOH and filtered, it can be concluded that both samples contain only IBN form B, see Figure 8.

**Figure 9:**
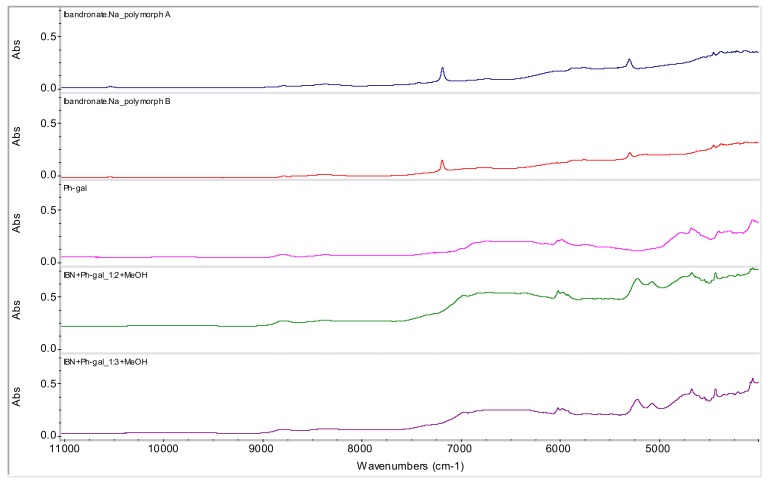
NIR spectra of ibandronate monosodium salt forms A and B, phenyl-β-d-galacto-pyranoside (Ph-gal) and spectra of potential co-crystals of IBN+Ph-gal in ratios 1:2 and 1:3 prepared by addition of MeOH and evaporation of liquid part at ambient temperature (samples **6** and **7**).

Figure 9 illustrates samples IBN+Ph-gal in ratios 1:2 (**6**) and 1:3 (**7**) after addition of MeOH and filtration of the obtained precipitate. After that, samples **6** and **7** were evaporated at ambient temperature. The same characteristic bands in the 5300-4800 cm^-1^ range can be observed in both Figure 7 and Figure 9. Based on this fact it can be concluded that the addition of MeOH does not influence the generation of a new unknown entity, because the same products were yielded with and without MeOH addition. Slow evaporation seems to be important, *i.e.* thermodynamically controlled crystal modification is probable. The presence of Ph-gal is fundamental for generation of the new entity.

**Figure 10:**
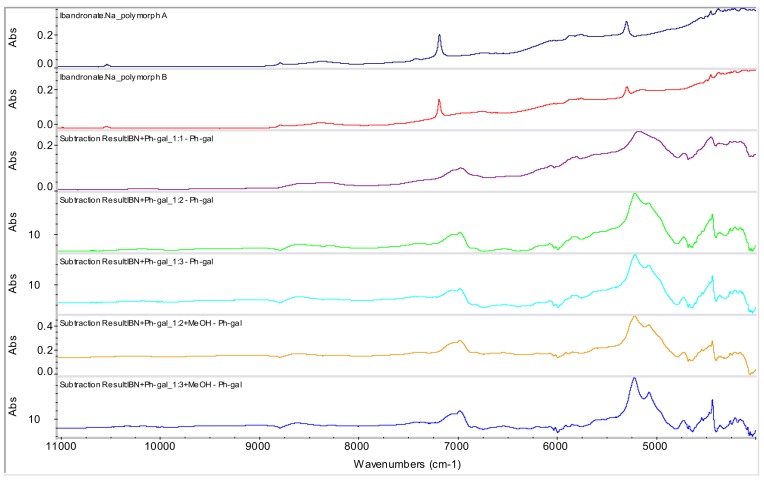
NIR spectra of ibandronate (IBN) monosodium salt forms A and B and subtracted spectra of potential co-crystals of IBN+Ph-gal in ratios 1:1, 1:2 and 1:3 (samples **1**-**3**) and potential co-crystals in ratios 1:2 and 1:3 prepared by addition of MeOH and evaporation of liquid part at ambient temperature (samples **6** and **7**).

The spectrum of phenyl-β-d-galactopyranoside was subtracted from the spectra of interesting samples **1**-**3**, **6** and **7**, and the subtraction results are documented in Figure 10. On the basis of these subtracted spectra it can be concluded that these prepared samples can be new entities (probably co-crystals), because these subtracted results are different from the spectrum of the starting material ibandronate mono-sodium salt form B, *i.e.,* the final product is not a simple mixture of ibandronate mono-sodium salt form B and phenyl-β-d-galactopyranoside.

**Figure 11:**
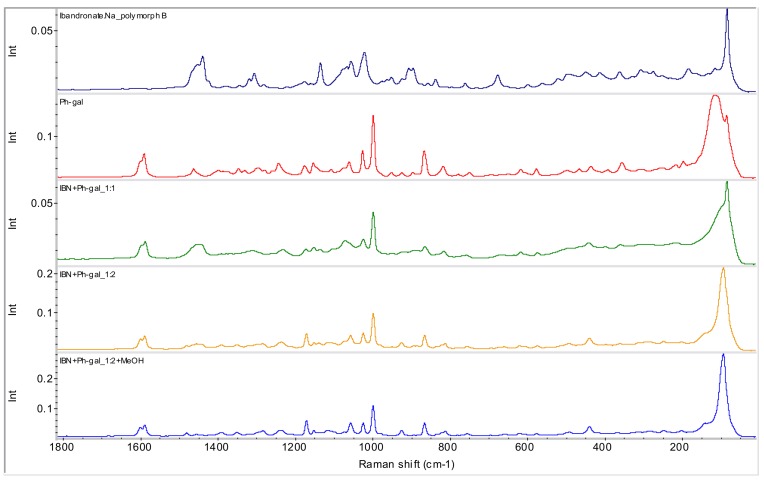
FT-Raman spectra of starting ibandronate monosodium salt form B, phenyl-β-d-galactopyranoside (Ph-gal) and spectra of their selected potential co-crystals (samples **1**, **2** and **6**).

**Figure 12:**
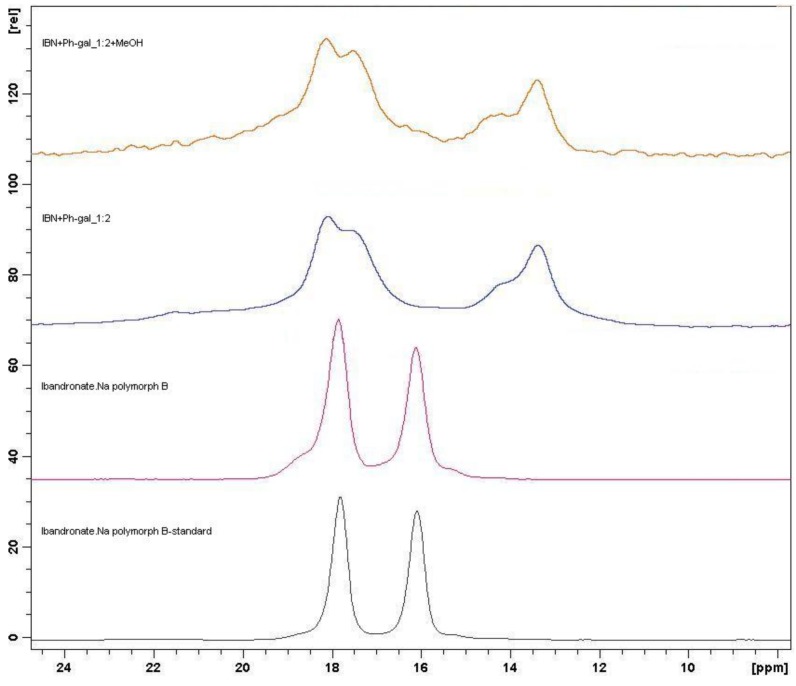
Comparison of ^31^P CP/MAS NMR spectra of starting ibandronate monosodium salt form B and spectra of selected potential co-crystals (samples **2** and **6**).

These samples were also characterized by means of the Raman spectrometry (see Figure 11) and ^31^P CP/MAS NMR spectroscopy (see Figure 12) for verification of this hypothesis. Both methods confirmed the presence of new entities.

Generally, based on the above discussed results, it can be concluded that the presence of phenyl-β-d-galactopyranoside and the thermodynamic condition (slow evaporation at ambient temperature) are fundamental for generation of new entities.

As bonds in co-crystals are formed by non-binding interactions (e.g., by *H*-bonds, ionic bonds, van der Waals forces (dispersion attractions, dipole-dipole, dipole-induced dipole interactions) and hydrophobic interactions), the steric arrangement of hydroxyl moieties on hexose skeletons seems to be important for co-crystal generation. Different interactions of ibandronate monosodium salt with phenyl-β-d-galactopyranoside compared to phenyl-β-d-glucopyranoside are probably caused by the opposite orientation of hydroxyl moiety in C_(4)_ in position 5 of the tetrahydropyran ring. In glucopyranosides this hydroxyl moiety is *trans*-oriented to pyran oxygen in position 1 of the tetrahydropyran ring, whereas the hydroxyl moiety in C_(4)_ of galactopyranosides is *cis*-oriented to pyran oxygen in position 1.

Contrary to the rest of tested agents, phenyl-β-d-galactopyranoside shows *cis*-orientation of hydroxyl moieties in C_(2)_ and C_(4)_ in positions 3 and 5 of the tetrahydropyran ring, *i.e.,* two hydroxyl moieties that possess *cis*-orientation with phenoxy moiety in C_(1)_ in position 2 of the tetrahydropyran ring. This fact is probably fundamental for interactions between ibandronate mono-sodium salt and phenyl-β-d-galactopyranoside, e.g., compared with structures of phenyl-β-d-glucopyranoside, methyl-β-d-galactopyranoside and naphthyl-β-d-galactopyranoside.

### 2.2. In vitro screening of absorption (PAMPA experiments)

Many low molecular weight drugs are absorbed through passive (or partially passive transport). The Parallel Artificial Membrane Permeability Assays (PAMPA) have become a very useful and quite cheap tool for predicting *in vivo* drug permeability and are well-suited as a ranking tool for the assessment of compounds with passive transport mechanisms. An absorption study of binary mixtures or final formulations is also possible on PAMPA plates. PAMPA can be used as an alternative approach to assess *in vitro* transcellular passive permeation [13].

The permeability of ibandronate (as a control sample) and samples **1**-**3**, **6** and **7** was tested. To retain interactions of both components in the solution it is recommended to increase the concentration of the counterion in the applied mixture [16]. Therefore samples **1**-**3**, **6** and **7** with the addition of sixfold quantity of phenyl-β-d-galactopyranoside to the co-crystal solution were also evaluated. The results of absorption study are shown in Table 2.

According to the above presented data (see Table 2), it can be stated that the highest absorption was expressed by ibandronate mono-sodium salt (the standard and/or the control sample) and by sample **1** with the lowest amount of phenyl-β-d-galactopyranoside. The following trend is evident: absorption decreases with increasing concentration of galactopyranoside.

## 3. Experimental

### 3.1. General

All reagents, excipients and solvents of analytical grade were purchased from Sigma-Aldrich. Ibandronate monosodium salt polymorph B used as the starting material is a standardized product of Eczacibaşi-Zentiva, Turkey. Near infrared spectra were recorded using Smart Near-IR UpDrift™, Nicolet™ 6700 FT-IR Spectrometer (Thermo Scientific, USA). The spectra were obtained by accumulation of 128 scans with 4 cm^-1^ resolution in the region of 12800-4000 cm^-1^. FT-Raman spectra were accumulated by a FT-Raman spectrometer RFS 100/S (Karlsruhe, Bruker, Germany). The spectra were obtained by accumulation of 256 scans with 4 cm^-1^ resolution in the back scattering geometry with the laser wavelength of 1064 nm. ^31^P CP/MAS NMR Spectra were recorded on Bruker AVANCE 500 MHz (Karlsruhe, Bruker, Germany). The ^31^P CP/MAS spectra were measured in 4 mm rotor at 10 kHz with 2 ms contact time. The ^31^P shift of NH_4_H_2_PO_4_ (0 ppm) was used as an external reference for ^31^P chemical shifts.

### 3.2. Generation of co-crystals

All the evaluated samples with ratios 1:1, 1:2 and 1:3 were prepared by means of dissolution of ibandronate monosodium salt (polymorph B) and the excipient in water, subsequently mixed and slowly evaporated at ambient temperature. To some samples with ratios 1:2 and 1:3 methanol was slowly added dropwise as anti-solvent. The solid precipitated compound was filtered and dried at ambient temperature and the remaining liquid part was slowly evaporated at ambient temperature. All generated solid compounds were subsequently characterized using the above mentioned spectroscopic methods. Particular preparations of the ibandronate and phenyl-β-d-galactopyranoside co-crystals in various ratios are described in Table 3 and Table 4.

### 3.3. In vitro screening of absorption (PAMPA experiments)

The permeability of generated co-crystals was evaluated *in vitro*, using a vertical PAMPA (Parallel Artificial Membrane Permeability Assay) system (BD GentestTMPre-Coated PAMPA Plate System, 96 wells, http://www.bdbeurope.com). The donor samples were prepared by dissolving the tested potential co-crystals in 0.01 M HCl, and than pH was adjusted by bicarbonate buffer to pH 7.4. The control sample was prepared in the same manner from ibandronate standards. A carbonate buffer saline (physiological solution) with pH 7.4 was used as a receptor phase. About 0.5 h before the experiment the PAMPA system was taken out from the freezer and warmed up to the ambient temperature. The receptor phase (200 μL/well) was pipetted into the upper wells. The donor phase was pipetted into the lower ones (300 μL/well). After the incubation time (5 h) 10 μL of the acceptor phase was taken from each well and mixed with physiological solution (990 μL). At least five determinations were performed. The results are summarized in Table 2.

Analysis of samples was performed using a LTQ Orbitrap Hybrid Mass Spectrometer (Thermo Electron Corporation, USA) in the negative mode ESI, SIM 318 m/z. A Waters XTerra^®^ MS C_18_ Direct Connect Column, 3.5 µm, 2.1 × 150 mm (Waters Corp., Milford, MA, USA) was used. The mixture of MeCN (HPLC grade, 80.0%) and H_2_O (HPLC – Mili-Q Grade, 20.0%) was used as a mobile phase. The total flow of the column was 0.2 mL/min. The results are summarized in Table 2.

## 4. Conclusions

Eleven gluco- and galactopyranoside derivatives were tested as counterions for generation of co-crystals with ibandronate monosodium salt. Seventy-seven samples were prepared. All samples were screened by FT-NIR spectrometry. NIR spectra of the prepared samples were compared with the starting materials, the subtraction results of the samples and the starting gluco- and galactopyranoside derivatives were calculated, and some new entities were predicted and checked by FT-Raman spectrometry and ^31^P CP/MAS NMR spectroscopy. Only phenyl-β-d-galactopyranoside yielded potential co-crystals with ibandronate probably due to *cis*-orientation of phenoxy moiety in C_(1)_ and hydroxyl moieties in C_(2)_ and C_(4)_ in positions 2, 3 and 5 of the tetrahydropyran ring. All the potential co-crystals characterized by all three spectroscopic methods were evaluated for their absorption using the PAMPA method. All the evaluated co-crystals of ibandronate and phenyl-β-d-galactopyranoside showed similar or relatively low absorption related to permeability of ibandronate, which is in conflict with our expectation. Probably, the higher amount of phenyl-β-d-galactopyranoside blocked pores of the artificial membrane, and this caused a decrease in absorption.

## Figures and Tables

**Table 1:** Prepared samples of ibandronate (IBN) and used sugar derivatives in ratios 1:1, 1:2 and 1:3 prepared by evaporation at ambient temperature, samples in ratios 1:2 and 1:3 prepared by methanol precipitation and samples in ratios 1:2 and 1:3 prepared by addition of MeOH and evaporation of liquid part at ambient temperature.

**Comp.**	**1:1**	**1:2**	**1:3**	**1:2**	**1:3**	**1:2**	**1:3**
				+MeOH precipitate		+MeOH (filtrate)	
**IBN+Glu**	B	B	B	B	B	B	B
**IBN+Trehalose**	B	B	B	B	B	B	B
**IBN+Glu acid lactone**	B	B	B	B	B	B	B
**IBN+Me-glu**	B	B	B	B	B	B	B
**IBN+3-*O*-Me-glu**	B	B	B	B	B	B	B
**IBN+Octyl-glu**	B	B	B	B	B	B	B
**IBN+Ph-glu**	B	B	B	B	B	A+B	A+B
**IBN+Gal**	B	B	B	B	B	B	B
**IBN+Me-gal**	B	B	B	B	B	B	B
**IBN+Phe-gal**	new	new	new	B	B	new	new
**IBN+Naph-gal**	B	B	B	B	B	A+B	A+B

α-d-glucose: Glu, d-(+)-trehalose, d-(+)-gluconic acid δ-lactone: Glu acid lactone, methyl-α-d-glucopyranoside: Me-glu, 3-*O*-methyl-α-d-glucopyranoside: 3-*O*-Me-glu, octyl-β-d-glucopyranoside: Octyl-glu, phenyl-β-d-glucopyranoside: Ph-glu, α-d-galactopyranoside: Gal, methyl-β-d-galactopyranoside: Me-gal, phenyl-β-d-galactopyranoside: Ph-gal, 2-naphthyl-β-d-galactopyranoside: Naph-gal.

**Table 2:** Concentration of ibandronate standard and co-crystal samples **1**-**3**, **6**, **7**, **1a**-**3a**, **6a** and **7a** in acceptor solutions of PAMPA plates. Data for PAMPA were calculated from 5 experiments.

Compound	Conc. [μg/mL]
IBN monosodium salt	2.4
IBN+Ph-gal 1:1 (**1**)	2.4
IBN+Ph-gal 1:2 (**2**)	2.1
IBN+Ph-gal 1:3 (**3**)	2.06
IBN+Ph-gal+MeOH 1:2 (**6**)	2.2
IBN+Ph-gal+MeOH 1:3 (**7**)	2.01
IBN+Ph-gal 1:1 (1:6) (**1a**)	<2
IBN+Ph-gal 1:2 (1:6) (**2a**)	<2
IBN+Ph-gal 1:3 (1:6) (**3a**)	<2
IBN+Ph-gal+MeOH 1:2 (1:6) (**6a**)	<2
IBN+Ph-gal+MeOH 1:3 (1:6) (**7a**)	<2

**Table 3:** Ibandronate (IBN) and phenyl-β-d-galactopyranoside (Ph-gal) in ratios 1:1, 1:2 and 1:3 (samples **1**-**3**).

Comp.	1:1		1:2		1:3	
	Amount [g]	Water [mL]	Amount [g]	Water [mL]	Amount [g]	Water [mL]
**IBN**	0.3493	3.3	0.3501	3.0	0.3505	3.0
**Phe-gal**	0.2477	2.0	0.5008	3.0	0.7228	3.5

**Table 4:** Ibandronate (IBN) and phenyl-β-d-galactopyranoside (Ph-gal) in ratios 1:2 and 1:3 with addition of methanol (samples **4**-**7**).

Comp.	1:2			1:3		
	Amount [g]	Water [mL]	MeOH [mL]	Amount [g]	Water [mL]	MeOH [mL]
**IBN**	0.3407	1.5	5.0	0.3627	2.0	5.0
**Phe-gal**	0.5003	3.0	5.0	0.7265	3.5	5.0
